# Infective larvae of *Anisakis simplex* (Nematoda) accumulate trehalose and glycogen in response to starvation and temperature stress

**DOI:** 10.1242/bio.040014

**Published:** 2019-03-15

**Authors:** Elżbieta Łopieńska-Biernat, Robert Stryiński, Małgorzata Dmitryjuk, Barbara Wasilewska

**Affiliations:** 1 Department of Biochemistry, Faculty of Biology and Biotechnology, University Warmia and Mazury in Olsztyn, Oczapowskiego 1A, 10-719 Olsztyn, Poland; 2 Department of Animal Anatomy and Physiology, Faculty of Biology and Biotechnology, University Warmia and Mazury in Olsztyn, Plac Łódzki 3, 10-727 Olsztyn, Poland

**Keywords:** Glycogen, Nematoda, Trehalose, Stress protection, *Anisakis simplex*

## Abstract

*Anisakis simplex* L3 larvae infect fish and other seafood species such as squid or octopi; therefore, humans consuming raw or undercooked fish may become accidental hosts for this parasite. These larvae are induced to enter hypometabolism by cold temperatures. It is assumed that sugars (in particular trehalose and glycogen) are instrumental for survival under environmental stress conditions. To elucidate the mechanisms of environmental stress response in *A. simplex*, we observed the effects of starvation and temperature on trehalose and glycogen content, the activity of enzymes metabolizing those sugars, and the relative expression of genes of trehalose and glycogen metabolic pathways. The L3 of *A. simplex* synthesize trehalose both in low (0°C) and high temperatures (45°C). The highest content of glycogen was observed at 45°C at 36 h of incubation. On the second day of incubation, tissue content of trehalose depended on the activity of the enzymes: TPS was more active at 45°C, and TPP was more active at 0°C. The changes in TPP activity were consistent with the transcript level changes of the TPP gene, and the trehalose level, while glycogen synthesis correlates with the expression of glycogen synthase gene at 45°C; this suggests that the synthesis of trehalose is more essential. These results show that trehalose plays a key role in providing energy during the thermotolerance and starvation processes through the molecular and biochemical regulation of trehalose and glycogen metabolism.

## Abstract (precis)

**Summary:** Carbohydrate metabolism in L3 of *A. simplex* is affected by temperature shock and by starvation. The metabolic shift from glycogen to trehalose suggests that blocking or silencing the trehalose synthesis pathway could be a limiting factor for Anisakis life and development.

## INTRODUCTION

*Anisakis simplex* is a parasitic aquatic nematode that colonizes crustaceans, cephalopods, fish and marine mammals. The occurrence of *Anisakis* nematodes has been reported in all major oceans and seas (Sakanari and McKerrow, 1989). The accidental intake of these nematodes, generally after the consumption of raw or undercooked parasitized fish, (herring, hake, horse mackerel and cod) can cause digestive disorders and/or allergies in humans (Valls et al., 2005; Audicana and Kennedy, 2008). *Anisakis* larvae produce proteolytic enzymes, penetrate the host's gastric and intestinal mucosa and cause mucosal infections which are referred as anisakiasis (Sakanari and McKerrow, 1990). According to the sanitary authorities of the USA and the EU, fish products should be cooked at 60°C for 10 min or longer to prevent infection. Fish products that are not intended for cooking or processing at temperatures higher than 60°C should be deep frozen at −20°C for 24 h or at −35°C for >15 h or at −23°C for a minimum of 7 days. Decapitation and evisceration of freshly caught fish and storage at low temperature until consumption are recommended (Audicana et al., 2002; Garcia et al., 2012; Nieuwenhuizen and Lopata, 2014). The regulations issued by the USA and EU sanitary authorities can be found online at http://www.fda.gov/ and https://europa.eu/european-union/index_en.

Trehalose and glycogen play important roles in free-living, entomopathogenic and parasitic nematodes exposed to thermal, oxidative and desiccation stress (Wharton et al., 2000; Grewal and Jagdale, 2002; Jagdale et al., 2005). For these reasons, carbohydrate metabolism in L3 larvae of *A. simplex* continues to attract the interest of researchers with the aim of determining new anthelmintics. The results of our previous study of L3 larvae of *A. simplex* (Łopieńska-Biernat et al., 2006), which do not feed and rely solely on external sources of energy, support analyses of the hypometabolic state of this larvae. The mechanisms which are implicated in the regulation of carbohydrate metabolism under stress and which ensure high survival have to be studied in greater detail.

Trehalose [α, D-glucopyranosyl-(1→1)-α, D-glucopyranoside] is a ubiquitous nonreducing disaccharide (Eastmond et al., 2002) in all kingdoms, excluding mammals (El-Bashiti et al., 2005). Living organisms rely on trehalose as a source of energy which stabilizes cell membranes, is responsible for the liquid-crystal phase of phospholipid bilayers, participates in protein folding and stabilizes native proteins (Singer and Lindquist, 1998; Silva et al., 2005; Zhu et al., 2010). Nematodes synthesize trehalose in two steps. In the first step, trehalose-6-phosphate synthase (TPS, EC 3.1.3.12) catalyzes the synthesis of trehalose-6-phosphate (T6P) from glucose-6-phosphate (G6P) and uridine diphosphate glucose (UDPG). In the second step, the dephosphorylation of T6P to trehalose is catalyzed by trehalose-6-phosphate phosphatase (TPP, EC 2.4.1.15) (Pellerone et al., 2003; El-Bashiti et al., 2005). Łopieńska-Biernat et al. (2014, 2015) demonstrated TPP and TPS activity and the expression of *tps* and *tpp* genes in *A. simplex*. The expression and activity of TPS and TPP under exposure to stress have never been studied in *A. simplex*. Trehalose undergoes hydrolytic and phosphorolytic degradation. During hydrolysis, trehalose is broken down by the enzyme trehalase (EC 3.2.1.28), whereas phosphorolytic decomposition is catalyzed by trehalose phosphorylase (EC 2.4.1.64) (Dmitryjuk and Żółtowska, 2003; Łopieńska-Biernat et al., 2007). It is believed that trehalose synthesis enzymes, TPS and TPP, encoded by *tps* and *tpp* genes belong to the family of heat-shock proteins (HSPs) (Jagdale et al., 2005). Silva et al. (2005) observed that TPS was more thermostable than TPP in *Thermus thermophilus* and determined the temperature optima at 98°C for TPS and 70°C for TPP. Thus, we assumed that TPS and TPP could be potential novel allergens of *A. simplex*. In *Caenorhabditis elegans*, HSPs act as chaperones and protect other proteins against denaturation (Plesofsky and Brambl, 1999; Wharton, 2002). When introduced to the *Heterorhabditis bacteriophora* genome, HSPs increased the nematode's thermal tolerance (Hashmi et al., 1998; Wharton, 2002). Parasites colonizing warm-blooded hosts – unlike cold-blooded ones – have to acclimate to a sudden increase in temperature. HSPs enable nematodes to reproduce at high temperatures (Wharton, 2003). The silencing of *tps* and *tpp* genes inhibits trehalose accumulation, increases sensitivity to temperature fluctuations and decreases thermal tolerance (Hottiger et al., 1994; Majara et al., 1996; Jagdale et al., 2005).

Glycogen is a branched glucose polymer synthesized by living organisms as polysaccharide, the main source of carbon and energy. Glycogen is the main reserve sugar in helminths (Żółtowska, 1995). In nematodes its concentration varies across developmental stages and environmental conditions. Glycogen is broken down during starvation under anoxic conditions to release glucose for the synthesis of trehalose which exerts protective effects (Liu et al., 2004; Possik et al., 2015). The presence of glycogen is a major determinant of animal survival in unsupportive environments.

Glycogen is synthesized by the enzyme glycogen synthase (GS, EC 2.4.1.11) which catalyzes the α-1,4-glycoside bond between UDP-glucose and the glycogen polysaccharide chain of minimum four glucose residues. In nematodes, glycogen is decomposed during phosphorylation and hydrolysis reactions. Hydrolytic degradation involves α-amylase (EC 3.2.1.1) and amylo-1,6-glucosidase (EC 3.2.1.33). During the phosphorylation step, glycogen phosphorylase (GP, EC 2.4.1.1) catalyzes the breakdown of glycogen to glucose-1-phosphate (G1P) (Karpiak, 1965; Żółtowska, 1995). Phosphorolytic decomposition is more energy efficient because the released G1P is already phosphorylated and cannot be diffused from cells (Stryer, 2000). Neither the expression of genes encoding the above enzymes nor their activities have been studied in *A.* *simplex* under exposure to stress.

Due to described protective mechanisms in nematodes, invasive L3 larvae of *A. simplex* and their harmful antigens (proteinase inhibitor, somatic paramyosin and tropomyosin or HSPs) are not always effectively eliminated under exposure to high or low temperatures, which implies that *Anisakis* infections are likely to occur in the future even if food safety regulations are observed (McClelland, 2002; Moneo et al., 2005; Sánchez-Monsalvez et al., 2005; Audicana and Kennedy, 2008; Pascual et al., 2010; Fæste et al., 2014; Carrera et al., 2016; Morsy et al., 2017; Sánchez-Alonso et al., 2017). During hypometabolism, when the larva goes into a type of stasis and can survive harsh conditions, trehalose and glycogen metabolism and the enzymes involved in their synthesis and breakdown are important. Penkov et al. (2015) and Seo et al. (2018) suggested that the biosynthesis of trehalose and glycogen is involved in the regulation of dauer formation. Importantly, limiting glycogen storage leads to a metabolic shift whereby glucose is stored as trehalose. Low levels of G6P and NADPH in the *C. elegans* are induced by trehalose synthesis in DAF-12 and DAF-16/FoxO transcription factors pathways. Thus, dauer development is controlled through the integration of carbohydrate metabolic fluxes and cellular redox potential.

Temperature and starvation for *A. simplex* are factors that we can use against the larvae and make harmless. However, it is important to maintain high-quality products in which these larvae can occur. Determination of which stress conditions are more harmful, and which enzyme activity and gene expression is more affected could possibly answer the question of how to more effectively fight *A. simplex* larvae and anisakiasis.

Accordingly, the aim of this study was to determine the content of trehalose and glycogen in *A. simplex* under exposure to thermal stress and starvation. The activity of TPS, TPP, trehalase, GP and α-amylase and the mRNA expression of *tpp*, *tps*, *tre*, *gs* and *gp* genes were also analyzed.

## RESULTS

### Trehalose and glycogen content in L3 larvae of *A. simplex* incubated in 0.65% NaCl at the temperatures: 0°C, 4°C, 10°C, 37°C and 45°C

#### Changes in trehalose and glycogen content

**Fig. 1.**
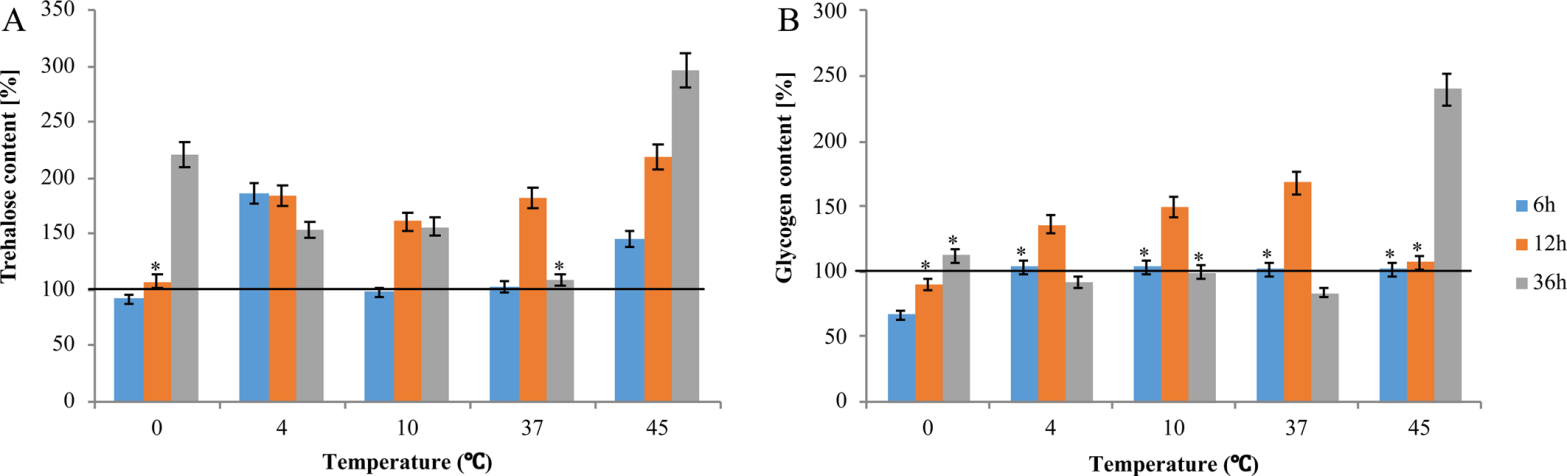
**Changes in trehalose and glycogen content at the 6, 12 and 36 h starvation of *A.simplex* larvae at different temperatures: 0°C, 4°C, 10°C, 37°C and 45°C.** Changes in trehalose content during starvation (A). Changes in glycogen content during starvation (B). Error bars represent means. Bars with asterisks indicate statistically insignificant differences (*P*<0.05). Data are presented as means±s.d. (*n*=3). Baseline value: 100%=24.28±8.80 [mg/g] for trehalose (A); 100%=66.47±10.3 [mg/g] for glycogen (B).

Trehalose content increased gradually relative to the baseline value throughout the experiment. The greatest differences were observed at 36 h of larval incubation at 0°C and 45°C. Trehalose concentration more than doubled at 0°C and increased threefold at 45°C (Fig. 1A). Glycogen content had already increased relative to the baseline value (100%) at 12 h of incubation at 4°C, 10°C and 37°C. Glycogen content increased by approximately 40% at 4 and 10°C and by approximately 60% at 37°C, relative to the baseline value (Fig. 1B). At a temperature of 45°C, glycogen content increased by 2.5-fold relative to the baseline value was observed at 36 h of incubation (Fig. 1B).

### Changes in the activity of anabolic and catabolic enzymes of trehalose metabolism in L3 larvae of *A. simplex* incubated in 0.65% NaCl at temperatures: 0°C, 4°C, 10°C, 37°C and 45°C

#### Changes in the activity of T6P synthase (TPS)

**Fig. 2.**
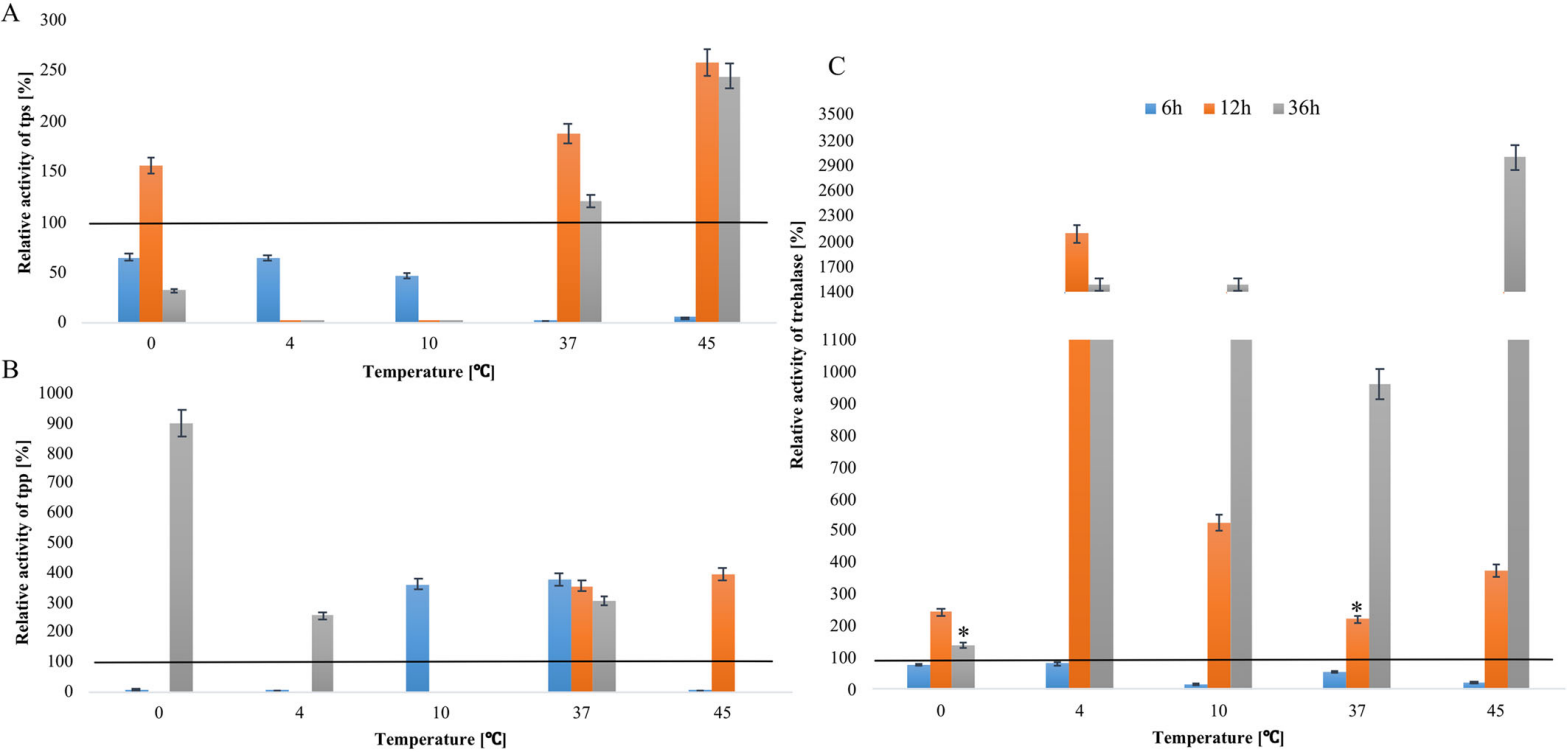
**Changes in TPS, TPP and trehalase activity at the 6, 12 and 36 h starvation of *A.simplex* larvae at different temperatures: 0°C, 4°C, 10°C, 37°C and 45°C.** Baseline value: 100%=0.192±0.024 [u/mg] for TPS (A); 100%=0.028±00.22 [u/mg] for TPP (B) and 100%=1.47±0.66 [u/mg] for TRE (C). Error bars represent means. Bars with asterisks indicate statistically insignificant differences (*P*<0.05). Data are presented as means±s.d. (*n*=3).

At 6 h of larval incubation, the activity of T6P synthase decreased at all temperatures relative to the baseline value. The greatest increase in T6P synthase activity was observed at 12 h of incubation at 0°C (approximately 1.5-fold), 37°C (twofold) and 45°C (threefold). However, TPS activity the above parameter decreased around 3.4-fold after incubation at 4°C and 10°C. At 36 h of incubation at 45°C, TPS activity increased by approximately 2.5-fold relative to the value measured in freshly isolated larvae (Fig. 2A).

#### Changes in the activity of T6P phosphatase (TPP)

The activity of T6P phosphatase decreased at 6 h of incubation at most temperatures relative to the baseline value. Enzyme activity increased around 3.5-fold only at 10°C and 37°C. Enzyme activity also was not observed in larvae fasted for 12 h at 0°C, 4°C and 10°C. The activity of T6P phosphatase increased approximately fourfold only in larvae incubated at 37 and 45°C. Very high levels of TPP activity were observed at 36 h of incubation at 0°C (ninefold). A 2.5-fold increase in TPP activity was noted at 4°C, and a fourfold increase was observed at 37°C (Fig. 2B).

#### Changes in trehalase activity

At 6 h incubation of *A. simplex* larvae, trehalase activity decreased at all temperatures relative to the baseline value. The greatest increase in trehalase activity was noted at 12 h of incubation at 4°C (approximately 20-fold) and at 36 h of incubation at 45°C (approximately 30-fold) (Fig. 2C).

### Changes in the activity of catabolic enzymes of glycogen metabolism in L3 larvae of *A. simplex* incubated in 0.65% NaCl at the temperatures: 0°C, 4°C, 10°C, 37°C and 45°C

#### Changes in glycogen phosphorylase activity (% gp)

**Fig. 3.**
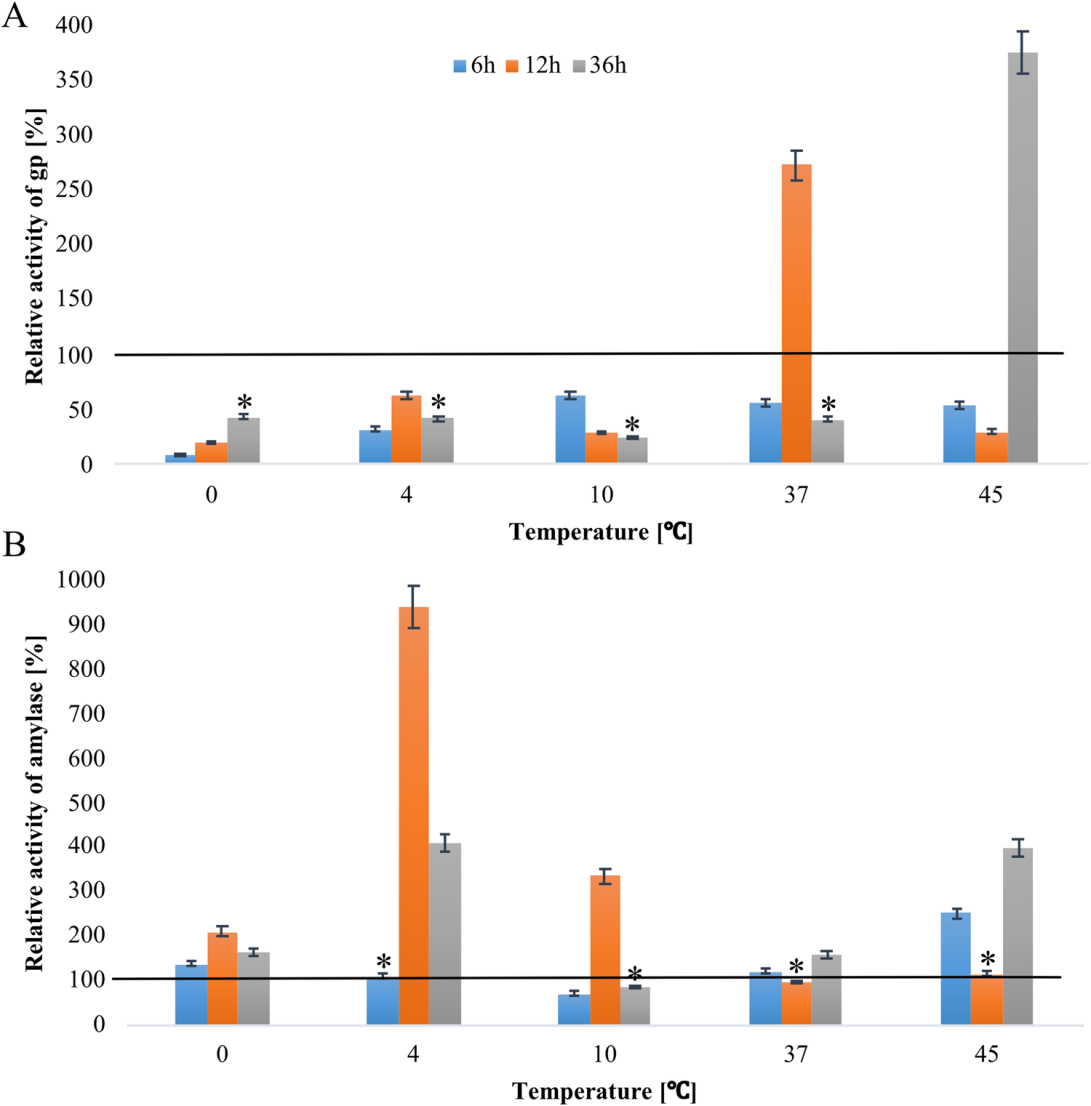
**Changes in α-amylase and glycogen phosphorylase activity at the 6, 12 and 36 h starvation of *A.simplex* larvae at different temperatures: 0°C, 4°C, 10°C, 37°C and 45°C.** Baseline value: 100%=19.4±5.49 [u/mg] for phosphorylase glycogen (A); 100%=2.42±0.4 [U/mg] for α-amylase (B). Error bars represent means. Bars with asterisks indicate statistically insignificant differences (*P*<0.05). Data are presented as means±s.d. (*n*=3).

The activity of glycogen phosphorylase at the beginning of the experiment was determined at 19.4 U/mg of protein (Fig. 3A). The analyzed parameter decreased at 6, 12 and 36 h of incubation at most tested temperatures, excluding 37°C and 45°C (Fig. 3A). The activity of the studied enzyme increased 2.5-fold at 12 h at 37°C, and the greatest increase (3.5-fold) was noted at 36 h of incubation at 45°C (Fig. 3A).

#### Changes in amylase activity

At 6 h of incubation, only a minor increase in amylase activity was noted at 37°C, whereas a 2.5-increase was observed at 45°C. At 12 h of incubation, amylase activity was similar at the 37 and 45°C to the baseline value, whereas at 4 and 10°C, ninefold and threefold, respectively. A visible increase in amylase activity was noted at 36 h of incubation at 4°C and 45°C (fourfold) (Fig. 3B).

### The expression of trehalose-6-phosphate synthase, phosphatase and trehalase mRNA relative to baseline values

**Fig. 4.**
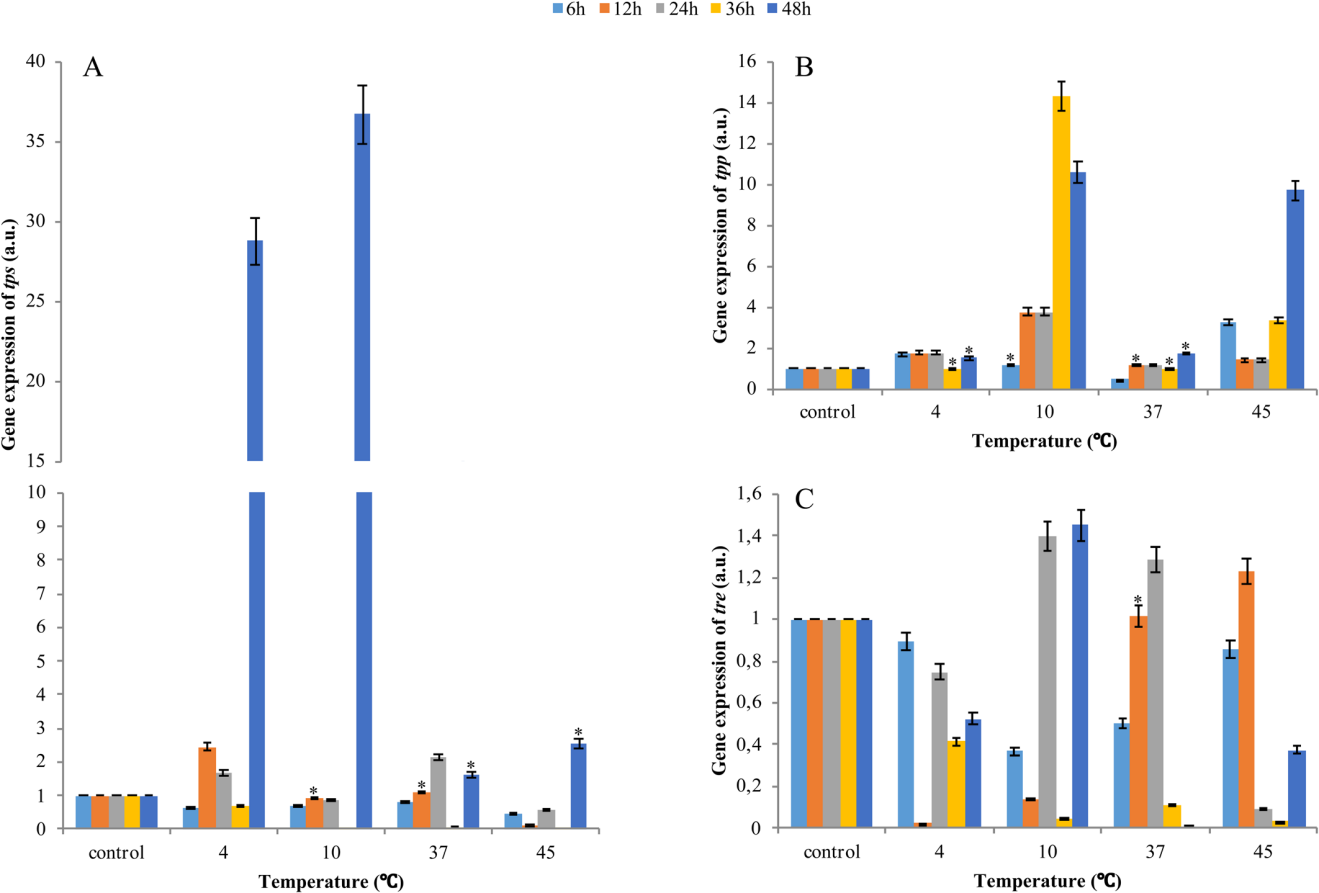
**Quantitative mRNA expression of trehalose metabolism genes at the 6, 12, 36 and 48 h starvation of *A.simplex* larvae at different temperatures: 4°C, 10°C, 37°C and 45°C.** mRNA expression of *tps* gene (A); mRNA expression of *tpp* gene (B); mRNA expression of *tre* gene (C). Error bars represent means. Bars with asterisks indicate statistically insignificant differences (*P*<0.05). Data are presented as means±s.d. (*n*=3).

TPS and TPP mRNA was expressed in all groups. However, differences in the expression of *tps* and *tpp* genes were noted during *in vitro* incubation of *A. simplex* (Fig. 4A,B). The mRNA expression of TPS decreased at 6 h of incubation at all temperatures. The observed differences between means were statistically significant in all cases (Fig. 4A). The expression of the *tps* gene at 12 h of incubation increased only at 4°C (twofold) relative to the baseline value. At 48 h, the expression of the *tps* gene increased significantly at 4°C, 10°C and 45°C. The differences between means were statistically significant in all groups (Fig. 4A). The expression of the *tpp* gene encoding the anabolic enzymes of trehalose was higher in all groups relative to the baseline value, excluding at 36 h of incubation at 4°C and 37°C. The above parameter increased 14-fold and ninefold at 36 and 48 h of incubation at 10°C and 45°C, respectively. The differences between mean values in the control (baseline value) and experimental groups were statistically significant (Fig. 4B).

The expression of the gene encoding trehalase also fluctuated during larval incubation (Fig. 4C). mRNA expression levels throughout the entire experiment were closer to the baseline value, and only a minor increase was observed at 12, 24 and 48 h of incubation at 10°C, 37°C and 45°C. The differences between mean values in the control (baseline value) and experimental groups were statistically significant (Fig. 4C).

### The expression of glycogen synthase and phosphorylase mRNA relative to baseline values

**Fig. 5.**
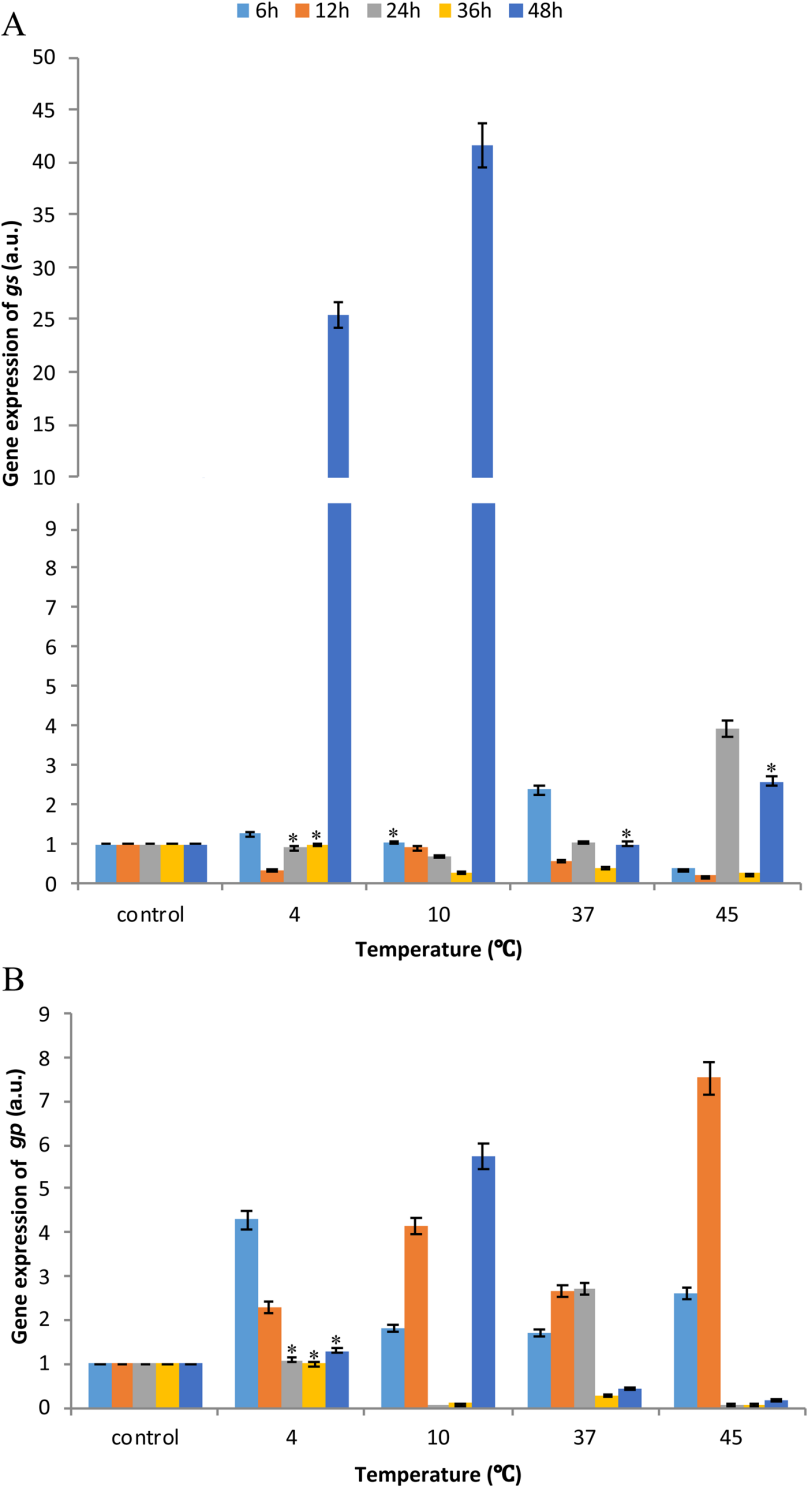
**Quantitative mRNA expression of glycogen metabolism genes at the 6, 12, 36 and 48 h starvation of *A. simplex* larvae at different temperatures: 4°C, 10°C, 37°C and 45°C.** mRNA expression of *gs* (A); mRNA expression of *gp* (B)*.* Error bars represent means. Bars with asterisks indicate statistically insignificant differences (*P*<0.05). Data are presented as means±s.d. (*n*=3). Baseline referred to the control (value=1).

GS and GP mRNA (gs and gp) was expressed in all temperature groups. However, differences in the expression of *gs* and *gp* genes were noted during *in vitro* incubation of *A. simplex* (Fig. 5). In most groups, the mRNA expression of GS decreased at 12, 24 and 36 h of incubation relative to the baseline value. An increase in expression was observed at 48 h of incubation. Significant differences were noted at 4°C (23-fold increase), 10°C (40-fold increase) and 45°C (threefold increase) relative to the baseline value. The observed differences between means were statistically significant in all cases (Fig. 5A).

The expression of the gene encoding GP also fluctuated during larval incubation (Fig. 5B). The greatest changes in the expression of the *gp* gene were observed at the beginning of incubation. Significant variations (2.5- to fourfold) were noted at 6 h of incubation at all temperatures. Similar values were noted at 12 h of incubation at all temperatures, and the most profound increase (7.5-fold) was observed at 45°C. The expression of the *gp* gene decreased during continued incubation at the most temperatures (Fig. 5B).

## DISCUSSION

Parasitic nematodes have developed numerous physiological and biochemical adaptive mechanisms supporting survival under adverse environmental conditions, such as thermal and osmotic shock, starvation and unfavorable pH (Wharton et al., 2000; Erkut et al., 2013). The molecular and biochemical bases of these adaptive traits have to be identified to expand our knowledge about parasites and their defense mechanisms.

Trehalose and glycogen metabolism contributes to the survival at the dauer stage of *C.* *elegans*. Dauer larvae are broadly used as a paradigm for studying hypometabolism (Erkut and Kurzchalia, 2015). This larval stage is resistant to a variety of stressors (e.g. starvation, desiccation, extreme temperatures, toxins) and can survive up to several months in laboratory conditions (Holt, 2006; Wang et al., 2009). The extended longevity of dauer larvae appears to be partly due to the increased expression of stress resistance genes, metabolic changes and insulin-like signaling (Olsen et al., 2006). The dauer development stage is thought to correspond to the infective stage found in many parasitic nematodes (Ogawa et al., 2009). Therefore, it seems interesting to know and compare the metabolism of infective L3 larva of *A. simplex.* Importantly, during dauer formation, limiting glycogen storage leads to a metabolic shift whereby glucose is stored as trehalose (Penkov et al., 2015). That is why, when *A. simplex* is changing its host, it is constantly exposed to harsh temperature conditions and starvation. The trehalose and glycogen metabolism are interconnected during environmental stress conditions and this link implies that the metabolism of both sugars is mutually regulated (metabolic shift from glycogen to trehalose) (Seo et al., 2018). Therefore, we described trehalose and glycogen metabolism in those two stress conditions (temperature and starvation).

Two sugars, trehalose and glycogen, were detected in the infective larvae of *A. simplex* (Łopieńska-Biernat et al., 2006). In unstable environments, nematodes accumulate large quantities of glycogen, the main reserve sugar that guarantees survival during prolonged starvation (Fairbairn and Passey, 1957; Żółtowska, 1995; Womersley and Higa, 1998). The trehalose content of freshly isolated L3 larvae of *A. simplex* was threefold lower than glycogen content (Fig. 1). In adult stages of parasitic nematodes such as *Ascaris suum*, *Trichinella spiralis*, *Pseudoteranova decipiens*, *Cystidicola farionis* larvae, *Hystherotylacium aduncum*, and free-living *Turbatrix aceti*, *Panagrellus redivivus*, and *Ditylenchus myceliophagus*, glycogen content usually exceeds trehalose levels, and is accumulated at the expense of trehalose (Von Brand, 1979; Womersley and Smith, 1981; Womersley et al., 1982; Żółtowska et al., 2002). In this study, under starvation stress, the trehalose content of *A. simplex* larvae was two- to four-times higher than glycogen content relative to the baseline value (Fig. 1). Similar results were reported by Behm (1997) who observed that trehalose was synthesized at the expense of glycogen. Glycogen levels can remain stable, whereas the concentration of trehalose, the circulating sugar in the dauer stage of *C. elegans* during anaerobic respiration, can fluctuate (McElwee et al., 2004; Braeckman et al., 2009). During prolonged fasting, glycogen is metabolized, whereas trehalose is synthesized. The above mechanism was identified in *A. simplex* larvae, where trehalose concentration increased two-times, whereas 30% change in glycogen content were noted only at 12 h (Fig. 1). In infective *A. simplex* larvae incubated at 36 h, an increase in glycogen levels was accompanied by a rise in trehalose content. Glycogen concentration also decreased with a drop in trehalose content at 6 and 12 h, which could be attributed to starvation (Fig. 1).

The negative correlation observed by Behm (1997) between trehalose and trehalase here was noticed only after 6 h of incubation at 4°C and 45°C. In *A. simplex*, prolonged starvation (36 h at 45°C) did not compromise trehalose content (Fig. 1 versus Fig. 2C). Similar correlations were reported in *S. cerevisiae* and *S. pombe*, where shock induced an increase in the activity of trehalase and TPS (Neves et al., 1991). Trehalose is a reserve sugar, and according to Behm (1997), it is the first compound to be utilized during fasting. A high concentration of trehalose can also be attributed to the activity of trehalose phosphorylase which synthesizes this disaccharide (Elbein et al., 2003; Dmitryjuk and Żółtowska, 2004).

In the present study, α-amylase activity was higher than the activity of glycogen phosphorylase at most temperatures (excluding at 12 and 36 h of incubation at 37°C and 45°C, respectively), which indicates that glycogen was broken down by enzymatic hydrolysis (Fig. 3). Similar observations were made by Karpiak and Iwanowski (1965) that α-amylase is responsible for the degradation of glycogen. Our findings are consistent with the results reported by Mohamed (2004) in *Heterorhabditis bacteriophora*, where the activity of α-amylase decreased with a drop in glycogen levels. In the present study, an increase in glycogen levels was also accompanied by an increase in α-amylase activity (Fig. 1 versus Fig. 3). The content of glycogen and mRNA expression of glycogen synthase intensified glycogen synthesis, which indicates that more glycogen was produced than hydrolyzed (Fig. 1 versus Fig. 5).

Nematodes that have acclimated to low temperatures are also characterized by higher levels of trehalose and higher survival rates (Wharton et al., 2000). In the life cycle of selected parasitic nematodes, the response to thermal shock plays a key role in survival and adaptation to a new environment. The filarial nematode *Brugia parangi* migrates from the poikilothermic mosquito vector (body temperature of 28°C) to homoeothermic vertebrates (body temperature of 37°C). In *B. parangi*, this change induces the synthesis of HSPs as a standard response to thermal shock (Thompson et al., 1996). In *H. bacteriophora* invasive entomopathogenic nematode, HSPs synthesis is an adaptive strategy in a changing environment (Selvan et al., 1996).

**Fig. 6.**
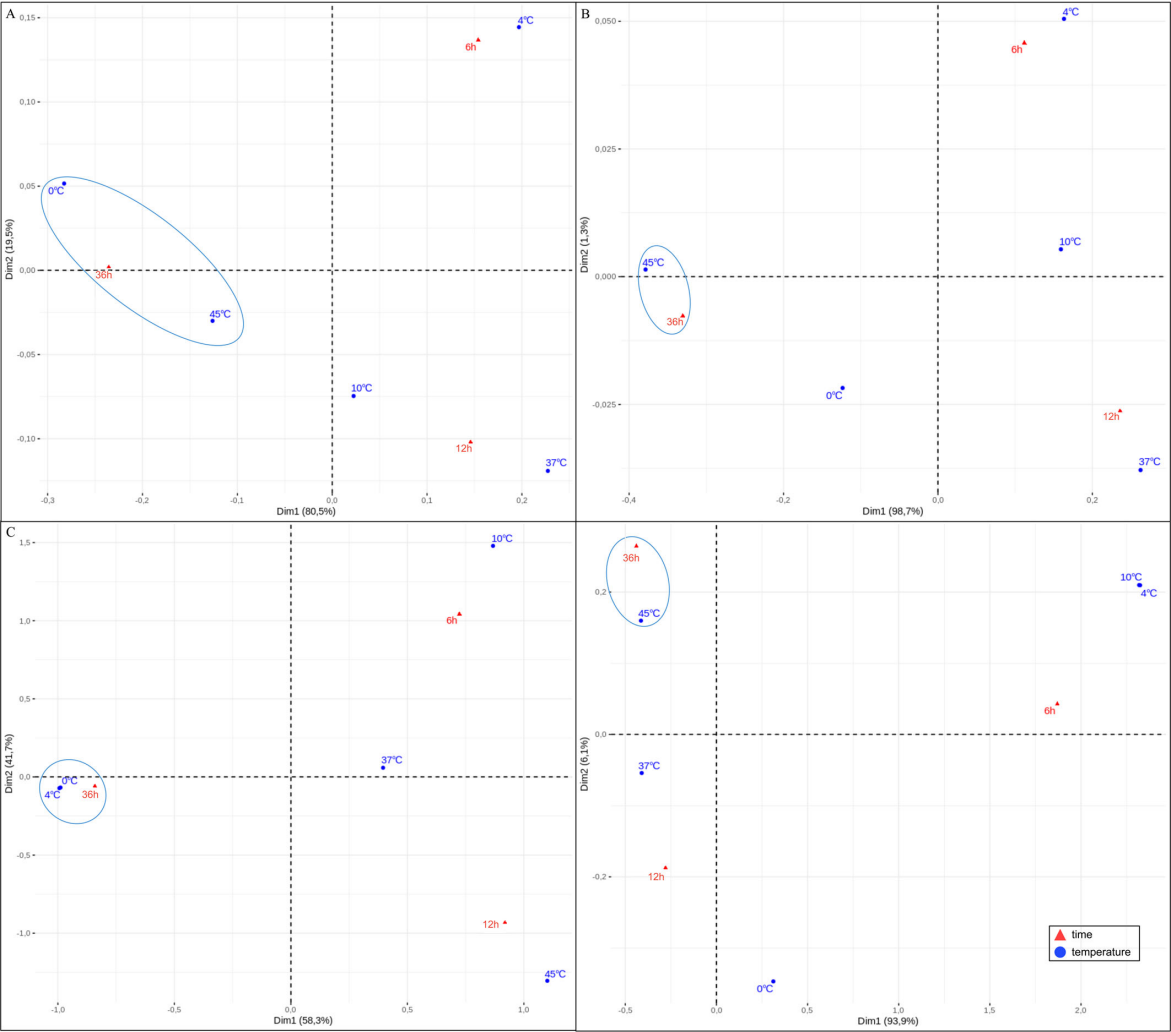
**The correspondence bi-plot of time and temperature of cultivation of *A. simplex* during thermal stress and starvation.** The relationship of time and temperature on trehalose (A) and glycogen content (B). The relationship of time and temperature on tpp (C) and tps activity (D). The closer the points are on the plot, the more correlated the relationship is.

The greatest differences in trehalose content were observed at 36 h of incubation of *A. simplex* larvae at 0°C and 45°C. Trehalose levels doubled at 0°C and tripled at 45°C relative to the baseline value (Fig. 1A). The correspondence analysis (CA) showed a high relationship between time (36 h) and extreme temperatures (0°C and 45°C) in respect to trehalose content (Fig. 6A). A similar relationship between temperature and trehalose concentration was observed in *Pseudoterranova decipiens*, a nematode of the family Anisakidae. Trehalose content doubled at low temperature, increased threefold at 37°C and sixfold at 45°C relative to room temperature (Stormo et al., 2009). Entomopathogenic nematodes were also found to accumulate trehalose in response to thermal stress (Jagdale and Grewal, 2003). In a study by Jagdale et al. (2005), trehalose content and the activity of T6P synthase increased considerably in entomopathogenic nematodes exposed to both high (35°C) and low (1°C and 10°C) temperatures. Similar observations were made in *A. simplex* larvae only at high temperatures when the activity of T6P synthase increased around threefold at 36 h of incubation at 45°C. At 12 h of incubation at 0°C, TPS activity increased to only 50% of the baseline value. However, the activity of TPP increased ninefold under exposure to 0°C (Fig. 2). The activity of TPP was correlated with its mRNA expression and trehalose content, which further emphasizes the important role of this enzyme (r=0.675) (Fig. 1 versus Figs 2,4). In *A. simplex* larvae, cold stress (0°C) induced more than a twofold increase in trehalose content, a 50% increase in TPS activity, and an ninefold increase in TPP activity (Fig. 1 versus Fig. 2). The correspondence analysis showed the highest relationship between time and temperature, low for TPP (Fig. 6C) and high temperature for TPS (Fig. 6D). Trehalose synthesis enzymes, TPS and TPP, which are encoded by *tps* and *tpp* genes probably belong to the HSP family (Jagdale et al., 2005). In our study, the activity of TPS and TPP at 45°C seems to confirm the above hypothesis (Fig. 2A,B). In parasitic nematodes *Brugia malayi*, *Toxocara canis* and *Ancylostoma ceylanicum* (Kushawaha et al., 2011; Cross et al., 2017) and free-living *C. elegans* (Kormish and McGhee, 2005), TPP enhances survival by regulating trehalose synthesis.

Parrou et al. (1997) observed that *S. cerevisiae* accumulated glycogen and trehalose and that trehalose levels were high only under exposure to thermal stress. Both sugars were accumulated by msn2/msn4 double mutants when the transcription of glycogen synthase (GSY2) and T6P synthase (TPS1) genes was induced, whereas no such changes were observed in wild yeast strains. Under exposure to stress, yeast utilize energy reserves to grow and reinstate glycogen and trehalose deposits. In the present study, L3 larvae of *A. simplex* synthesized trehalose and glycogen at the expense of endogenous energy reserves at incubation in 45°C (Fig. 1). The accumulation of trehalose and glycogen at higher temperatures was accompanied by an increase in the activity of sugar synthesizing enzymes and an increase in the expression of trehalose and glycogen synthesis genes (Fig. 1 versus Figs 2, 3, 4, 5). The above could indicate that transcription factors are activated only after prolonged exposure to stress at significantly higher temperature.

In the present study, the changes in the glycogen content of L3 larvae of *A. simplex* were intensified with an increase in incubation temperature. The above can be attributed to the fact that the basal metabolic rate increases with temperature. L3 larvae were more mobile at a temperature of 37°C, which could have led to greater depletion of glycogen reserves. Glycogen concentration decreased at 6 and 12 h of incubation at 37°C, whereas the highest glycogen content was noted at 36 h of incubation at 45°C (Fig. 1). The correspondence analysis also showed a high relationship in that conditions (Fig. 6B). Contrary results were reported in a study by Castro and Fairbairn (1969) where glycogen levels in *T. spiralis* decreased already after 6 h of incubation and continued to dwindle during continued exposure to high temperature.

The results relating to the mutual regulation of trehalose and glycogen concentrations are not conclusive, which is why the metabolism of both sugars was analyzed. Changes in the content of both sugars proceed differently under exposure to thermal stress and starvation at the same time. Under exposure to both stressors, nematodes metabolize glycogen as a source of energy and store trehalose as a protective molecule. These observations point to the mutual regulation of glycogen and trehalose synthesis and breakdown pathways.

The results of this study indicate that *A.* *simplex* nematodes synthesize and accumulate significant quantities of trehalose under exposure to thermal stress during starvation. Metabolism of trehalose activity is determined by trehalase, TPS and TPP, which are activated at low and high temperatures. Trehalose increases tolerance to both extreme temperatures (0°C and 45°C), whereas glycogen is the main sugar to be utilized by fasting *A. simplex* larvae exposed to high temperature; this suggests that the synthesis of trehalose is more essential. Our results suggest that the metabolic pathways of both glucose sources, trehalose and glycogen, could be activated in *A. simplex* infective larvae to maximize their survival. Trehalose and glycogen metabolism is probably regulated at the transcriptional and post-translational level, similarly to the hypometabolic state in the dauer stage. The metabolic shift from the glycogen to trehalose suggest that blocking or silencing the trehalose synthesis pathway, could be a limiting factor for *Anisakis* life and development.

Further research into the metabolism of *A.* *simplex* larvae is needed to determine trehalose and glycogen mutual signaling pathways, transcription factors and genes encoding trehalose and glycogen metabolism, and whether trehalose synthesis is also correlated with signaling pathways regulating dauer formation.

## MATERIALS AND METHODS

### Nematodes

L3 larvae of *A. simplex* were isolated from fresh Baltic herring (*Clupea harengus membrans*) cleaned of host tissue residues, washed several times in 0.65% NaCl and immediately cultivated. 15 individuals of L3 larvae of *A. simplex* were starved in 0.65% NaCl at five temperatures, 0°C, 4°C, 10°C, 37°C and 45°C, without any nutrients, in three independent sets at the same time. The second of the chosen temperatures, (4°C), corresponds to the temperature of sea water in which herrings (approximate temperature of cold-blooded animals) – the paratenic hosts of the parasite – stay during the spring season, while the temperature of 37°C corresponds to the body temperature of marine mammals – the ultimate hosts. Temperatures: 0°C is below, and temperatures 10°C and 45°C are above the host temperatures, tested as stress parameters. The content of trehalose and glycogen, and the activity and mRNA expression of trehalose and glycogen enzymes were determined in freshly isolated larvae and at the 6, 12, 24, 36 and 48 h of starvation at the above temperatures. During the experiment 1125 individuals of L3 of *A. simplex* were used (five temperatures×five times equals 25 experimental conditions; each condition×15 individuals×three replicates).

The baseline content of trehalose and glycogen and the baseline activity of trehalose and glycogen enzymes in larvae isolated from fresh fish were set at 100%.

### Preparation of extracts for a quantitative determination of saccharides and enzymatic analysis

L3 larvae were rinsed several times in 0.65% NaCl, dried on filter paper and weighed. They were homogenized manually in a glass homogenizer with 0.65% NaCl (1/10 w/v). The larvae were centrifuged at 800× ***g*** for 15 min at 4°C. The supernatant was used to determine the content of trehalose and glycogen and their enzymes.

### The determination of carbohydrate content and enzymatic activity assays

The glycogen content was measured by the micro-method developed by Sølling and Esmann (1975). Trehalose content was determined by high-performance liquid chromatography (HPLC). The samples for HPLC were first boiled for 5 min, diluted with about twice their volume of ethanol and centrifuged. The supernatants were desiccated at 50°C, dissolved in acetonitrile/deionized water (3:2, v/v) and filtered with nylon Micro-Spin Filter Tubes (Alltech Associates, USA). 20 µl of each specimen was injected into the Shimadzu SCL-10A system with the RID 10A refractive detector (Kyoto, Japan). A high-performance carbohydrate cartridge column (4.6×250 mm; Waters, the Netherlands) was eluted with a mixture of acetonitrile/degassed and deionized water (75/25%, 1 ml×min^−1^) and kept at 35°C during analysis. The concentrations of trehalose and glycogen were analyzed using Chromax 2005 software (POL-LAB; Warsaw, Poland). The content of the studied sugars was expressed in mg per g of wet tissue.

The activity of TPS (EC 2.4.1.15) was determined by the method proposed by Giaever et al. (1988), and TPP activity (EC3.1.3.12) was analyzed by the method described by Kaasen et al. (1994). Trehalose, the end product of TPS and TPP enzymatic reactions, was identified and quantified by HPLC as described above. One enzymatic unit [u] represented the quantity of enzyme that produced 1 mmol of trehalose at 37°C.

Trehalase (EC 3.2.1.28) activity was measured using a modified Dahlqvist method (1984) and was expressed in enzymatic units. One enzymatic unit [u] corresponded to the quantity of enzyme that produced 1 µmol of glucose at 37°C in 1 h. At the end of reaction, content of glucose was identified and quantified by HPLC as described above. The activity of glycogen phosphorylase was determined based on the enzyme's ability to synthesize glycogen, as described by Cori et al. (1955). Inorganic phosphate formed during the reaction was assayed by the micro technique proposed by Chen et al. and described by Keleti and Lederer (1974). One enzymatic unit [u] was equivalent to the amount of the enzyme required for the production of 1 µmol of glucose-1-phosphate (G1P) at 37°C in 1 h. The activity of α-amylase was determined using the method described by Caraway (1959), and it was expressed in activity units [U]. One unit was equivalent to the amount of the enzyme required for the degradation of 10 mg of starch in the iodine-starch reaction in L3 larvae incubated at 37°C (pH 6.0) for 2 h.

The activity of the analyzed enzymes was expressed relative to 1 mg of protein (Bradford, 1976).

### Real-time polymerase chain reaction

Total RNA extraction was performed with the Total RNA Mini Kit according to the manufacturer's protocol (A&A Biotechnology, Poland). RNA was quantified spectrophotometrically (A260 nm), and its quality was determined by gel electrophoresis on 1.5% agarose containing 0.01% ethidium bromide.

First-strand cDNA was synthesized with 2 μg of total RNA, 10 mM of a species-specific reverse primer (Łopieńska-Biernat et al., 2015) and reverse transcriptase (TranScriba Kit, A&A Biotechnology, Poland). Quantitative Real-time PCR (qRT-PCR) was performed using SYBR Green Taq PCR-MIX (A&A Biotechnology, Poland) according to the manufacturer's protocol. A quantity of 20 μl of the reaction solution contained 1 μl of the template (1:10 dilution of cDNA product), 10 μl of SYBR Green Taq PCR-MIX (2×), 1 μl of 10 μM of each primer (Table 1), 6.6 μl of water, and 0.4 μl of ROX Reference dye II. qRT-PCR was performed in the Applied Biosystems 7500 Fast Real Time PCR System (Life Technologies, USA). The following thermal cycling conditions were applied: 10 min at 95°C followed by 38 cycles of 20 s at 95°C, 20 s at 55°C, and 30 s at 72°C. Relative transcript levels at each time point were analyzed based on mean values and standard deviation with the use of the 2^–ΔΔCT^ method proposed by Livak and Schmittgen (2001). The data were presented as the fold change in gene expression normalized to an endogenous reference gene 18S rRNA (ribosomal; housekeeping gene U81575) and relative to the untreated control [relative quantification (RQ)=1; start group]. Transcript levels were determined in AB analytical software (7500 v2.0). Each assay was performed in triplicate. Melting curves were constructed after amplification.

**Table 1.**
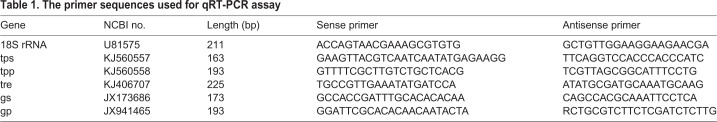
**The primer sequences used for qRT-PCR assay**

### Statistical analysis

Data were expressed as means±standard deviation by one-way ANOVA in the Statistica 12 software (StatSoft Inc., Tulsa, Oklahoma, USA). Differences between means were assessed by Tukey's honestly significant difference (HSD) test. *P*-values below 0.05 (*P*<0.05) were considered statistically significant. The correlations between the relative activity of TPS, TPP and GP and their mRNA expression and content of carbohydrates over time were determined by calculating Pearson's *r*. Additionally, correspondence analysis (CA) was done using the R commander software. The closer the points are on the plot, the more correlated the relationship is. The results were presented as mean values from three independent assays.
